# Supplementary figures and images for: Lipids Regulate Export of Lysosomal Enzymes from the Endoplasmic Reticulum
Source: bioRxiv. 2026 Apr 17:2026.04.16.719038. Preprint. [Version 1] doi: 10.64898/2026.04.16.719038 (PMC13104869; doi:10.64898/2026.04.16.719038)

## Supplementary Figure 2

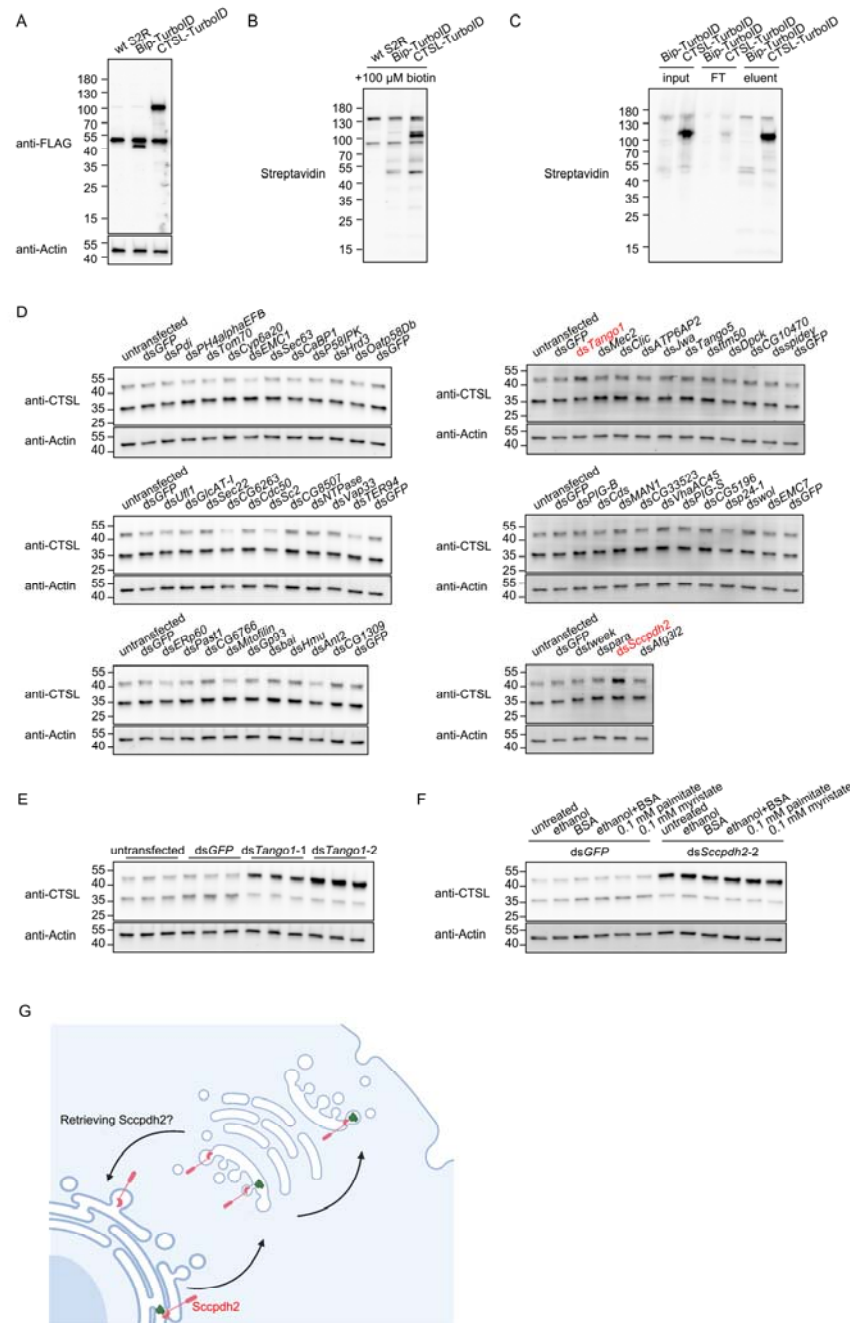

Supplement: 1 — Supplementary Figure 1. Arf1/COPI regulates CTSL transport independently of fatty acids. A. Western blot analysis of CTSL in cells following depletion of known CTSL-interacting proteins. B. Western blot analysis of CTSL in Arf1 or AlphaCOP knockdown cells supplemented with 0.1 mM palmitate (C16:0) or myristate (C14:0). Supplementary Figure 2. Proximity labeling identifies novel regulators of CTSL transport. A. Western blot analysis of FLAG-tagged BiP-TurboID and CTSL-TurboID expression. B. Western blot analysis of biotinylated proteins in cells transfected with plasmids encoding BiP-TurboID and CTSL-TurboID. Cells were treated with 100 μM biotin for 2 hours to allow proximity-dependent biotinylation. C. Western blot analysis of biotinylated proteins in the pulldown samples. FT, flow-through. D. Western blot analysis of CTSL in following knockdown of candidate genes identified by proximity labeling. E. Western blot analysis of CTSL in Tango1 knockdown cells. Two different non-overlapping dsRNAs targeting Tango1 were used. F. Western blot analysis of CTSL in Sccpdh2 knockdown cells supplemented with 0.1 mM palmitate (C16:0) or myristate (C14:0). G. Model: Sccpdh2 functions as a cargo receptor to facilitate CTSL export from the ER to the Golgi, and its retrieval is required to sustain continuous CTSL trafficking. [file NIHPP2026.04.16.719038V1-supplement-1.pdf]
